# RAC1 inhibition reverses cisplatin resistance in esophageal squamous cell carcinoma and induces downregulation of glycolytic enzymes

**DOI:** 10.1002/1878-0261.12548

**Published:** 2019-07-27

**Authors:** Rui‐Jie Zeng, Chun‐Wen Zheng, Jing‐E Gu, Hai‐Xia Zhang, Lei Xie, Li‐Yan Xu, En‐Min Li

**Affiliations:** ^1^ Department of Biochemistry and Molecular Biology Shantou University Medical College China; ^2^ The Key Laboratory of Molecular Biology for High Cancer Incidence Coastal Chaoshan Area Shantou University Medical College China; ^3^ Institute of Oncologic Pathology Shantou University Medical College China

**Keywords:** chemoresistance, cisplatin, esophageal squamous cell carcinoma, glycolysis, RAC1

## Abstract

Development of chemoresistance remains a major challenge in treating esophageal squamous cell carcinoma (ESCC) patients despite treatment advances. However, the role of RAC1 in chemoresistance of ESCC and the underlying mechanisms remain largely unknown. In this study, we found that higher levels of RAC1 expression were associated with poorer prognosis in ESCC patients. Enhanced RAC1 expression increased cell proliferation, migration, and chemoresistance *in vitro*. Combination therapy using RAC1 inhibitor EHop‐016 and cisplatin significantly promoted cell viability inhibition, G2/M phase cycle arrest, and apoptosis when compared to each monotherapy. Mechanistically, glycolysis was significantly downregulated in the RAC1 inhibitor monotherapy group and the combination group via inhibiting AKT/FOXO3a signaling when compared to the control group. Moreover, the silencing of RAC1 inhibited AKT/FOXO3a signaling and cell glycolysis while the upregulation of RAC1 produced an opposite effect. In murine xenograft models, the tumor volume and the expression of glycolytic enzymes were significantly reduced in combination therapy when compared to each monotherapy group. Overall, our study demonstrates that targeting RAC1 with an inhibitor overcomes cisplatin resistance in ESCC by suppressing glycolytic enzymes, which provides a promising strategy for treatment of ESCC in clinical practice.

**Abbreviations**
ALDOA: aldolase A
CI: confidence interval
DDP: cisplatin
DFS: disease‐free survival
EdU: 5‐ethynyl‐2′‐deoxyuridine
EHOP: EHop‐016
ESCC: esophageal squamous cell carcinoma
GEF: guanine nucleotide exchange factor
GO: gene ontology
H&E: hematoxylin and eosin
HK1: hexokinase 1
IHC: immunohistochemistry
LDHA: lactate dehydrogenase A
mTOR: mammalian target of rapamycin
OS: overall survival
PI: propidium iodide
PKM: pyruvate kinase
qRT‐PCR: quantitative reverse transcription polymerase chain reaction
RAC1: Rac family small GTPase 1
RNA‐seq: RNA sequencing

## 1 Introduction

Esophageal cancer is the eighth most common cancer worldwide (Jemal *et al.*, 2011; Sakai *et al.*, 2013). Esophageal squamous cell carcinoma (ESCC), a malignancy of esophageal epithelial cells, accounts for about 90% of esophageal cancer cases over the world (Abnet *et al.*, 2017; Song *et al.*, 2017).

In clinical practice, surgery remains the gold standard for esophageal cancer treatment (Allum *et al.*, 2014). However, prognosis of esophageal cancer patients is poor with a 5‐year survival rate of around 25% among patients undergoing surgery alone because most of the patients are only symptomatic in the advanced stage (Allum *et al.*, 2014; D’Amico, 2007). In addition, approximately half of the patients who undergo esophageal resection will develop systemic or local recurrences (Wang *et al.*, 2014). As a result, chemotherapy is used in combination with traditional surgery to optimize therapeutic outcomes. Cisplatin is a widely used chemotherapeutic agent for esophageal cancer (Yu *et al.*, 2014). When combining surgery with neoadjuvant chemotherapy using cisplatin, the 5‐year survival rate of patients can be significantly raised (D’Amico, 2007). However, great variations are seen in the drug resistance of different esophageal cancer patients, and certain patients are prone to develop chemoresistance toward cisplatin and other chemotherapeutic drugs (Alfarouk *et al.*, 2015; Kihara *et al.*, 2001; Schilsky, 2010). The resistance that ensues in esophageal cancer patients will lead to treatment failure and death (Alfarouk *et al.*, 2015; Takashima *et al.*, 2008; Zhu *et al.*, 2013), which remains a major challenge in treating esophageal cancer patients. Consequently, reversing chemoresistance and enhancing therapeutic efficacy are of great importance in terms of esophageal cancer treatments.

RAC1, as a member of RHO family GTPase, can be activated by guanine nucleotide exchange factors (GEFs) to a GTP‐bound state or be inactivated by GTPase‐activating proteins to a GDP‐bound state (Cardama *et al.*, 2017; Um *et al.*, 2014). In its GTP‐bound state, RAC1 activates a broad spectrum of downstream pathways and is important in modulating various cellular processes, including metastasis, migration, invasion, and cytoskeletal reorganization (Kamai *et al.*, 2010; Myant *et al.*, 2013; Wang *et al.*, 2015). According to the Genotype‐Tissue Expression (GTEx) database, the expression level of RAC1 is the highest in the esophagus among all human organs (Pontén *et al.*, 2011). RAC1 was also described to regulate chemotherapeutic sensitivity. For example, in lung cancer, silencing of RAC1 is related to an increase in chemosensitivity (Chen *et al.*, 2011). However, in epidermoid carcinoma and liver carcinoma cells, downregulation of RAC1 results in an increase in cisplatin resistance (Shen *et al.*, 2004). These contradictory findings draw our attention to this field. To date, the role of RAC1 in chemoresistance of ESCC remains unclear, and the mechanisms by which RAC1 regulates chemoresistance are largely unknown.

Therefore, we explored the relationship between RAC1 and the prognosis of ESCC patients and further investigated the role of RAC1 in ESCC development and chemoresistance. Additionally, we examined the effectiveness and the associated mechanisms of combination therapy of cisplatin and RAC1 inhibitor for ESCC.

## 2 Materials and methods

### 2.1 Patients and samples

One hundred and six human ESCC tumor samples (mean age: 58.0 years; 79 males and 27 females) were obtained from Shantou Central Hospital between October 2007 and July 2009, with approval for experiments from the Ethics Committee of Shantou University Medical College. The patients without local or systemic treatment before surgery were eligible for this study. The data of patient characteristics and histological examinations were obtained from medical reports and/or confirmed by two independent pathologists. Follow‐up by routine visits was performed, and the information was reviewed from outpatient records. Written informed consent was obtained from all patients, and the study methodologies conformed to the standards set by the Declaration of Helsinki. Table S1 demonstrates the clinical information of the patients in detail.

### 2.2 Immunohistochemistry

Immunohistochemistry (IHC) was performed on human specimens and xenograft mouse tumors as described previously (Li *et al.*, 2015b) using anti‐RAC1 (1 : 200 dilution; Abcam, Cambridge, UK), anti‐Ki67 (ZM0166, ready‐to‐use; ZSGB‐BIO, Beijing, China), anti‐PKM (1 : 200 dilution; Santa Cruz Biotechnology, Santa Cruz, CA, USA), anti‐LDHA (1 : 200 dilution; Santa Cruz Biotechnology, Santa Cruz, CA, USA), and anti‐HK1 (1 : 200 dilution; Santa Cruz Biotechnology, Santa Cruz, CA, USA) antibodies according to the manufacturers’ instructions.

### 2.3 Cell lines and reagents

Three ESCC cell lines, namely KYSE150, KYSE510, and TE5, were used in our study. The culture of these cell lines was described in our study before (Jiang *et al.*, 2018; Zou *et al.*, 2016). The cells were maintained at 37 °C under a humidified atmosphere of 5% CO_2_ and 95% air.

Cisplatin and EHop‐016, which were purchased from Selleck Chemicals (Houston, TX, USA) and MedChem Express (Monmouth Junction, NJ, USA), were dissolved in dimethylformamide and dimethylsulfoxide for storage, respectively.

### 2.4 Transfection of siRNAs and plasmids

Three RAC1 siRNAs (siRAC1‐1, siRAC1‐2, and siRAC1‐3) and three FOXO3a siRNAs (siFOXO3a‐1, siFOXO3a‐2, and siFOXO3a‐3) were purchased from GenePharma (Shanghai, China). Subsequently, siRAC1‐3 (RAC1 target sequence: 5′‐CUAAGGAGAUUGGUGCUGUTT‐3′) and siFOXO3a‐2 (FOXO3a target sequence: 5′‐CGUGAUGCUUCGCAAUGAUTT‐3′) were selected for experiment due to their best silencing effect. HiPerFect Transfection Reagent (QIAGEN, Valencia, CA, USA) was used for siRNA transfections with the manufacturer’s protocols.

Total RNA was isolated from the KYSE150 cell line by TRIzol reagent (Invitrogen, Carlsbad, CA, USA), and cDNA was synthesized using a Reverse Transcription System (TaKaRa, Otsu, Japan). Full‐length human *RAC1* cDNAs (lacking the TAA stop condon and containing *EcoR*I and *Xho*I restriction sites) were amplified using a forward primer (5'‐TCACCTATCCGCAGGGTCTA‐3') and a reverse primer (5'‐TCGCTTCGTCAAACACTGTC‐3'). After purification of the PCR products, the *EcoR*I/*Xho*I fragment of RAC1 was subcloned into pcDNA3.1 vector (Invitrogen), with a FLAG epitope tag at the N terminus. Sequencing was used to validate the recombinant plasmids. The cells were transfected using Lipofectamine 3000 (Invitrogen) following the manufacturer's instructions.

### 2.5 Western blotting

Protein extraction and western blot were performed as described previously (Li *et al.*, 2015b). Anti‐RAC1 (1 : 250 dilution) antibody was acquired from Cytoskeleton, Inc. (Denver, CO, USA). Anti‐PKM (1 : 500 dilution), anti‐LDHA (1 : 500 dilution), anti‐ALDOA (1 : 500 dilution), and anti‐HK1 (1 : 500 dilution) antibodies were obtained from Santa Cruz Biotechnology. Anti‐phospho‐AKT (Ser473) (1 : 1000 dilution), anti‐AKT (1 : 1000 dilution), anti‐phospho‐FoxO1 (Thr24)/FoxO3a (Thr32) (1 : 1000 dilution), anti‐FOXO3A (75D8) (1 : 1000 dilution), anti‐phospho‐S6 (Ser240/244) (1 : 1000 dilution), and anti‐S6 (5G10) (1 : 1000 dilution) antibodies were purchased from Cell Signaling Technology (Danvers, MA, USA).

### 2.6 Cell viability assay

For examination of cell proliferation ability, the assays were conducted as described before (Li *et al.*, 2015a). Briefly, transfected cells were first starved in a serum‐free medium for 12 h and further reseeded into 96‐well plates at an initial density of 7000 cells per well. After 0, 24, and 48 h, cell proliferation assays were performed using CellTiter 96 Aqueous One Solution Cell Proliferation Assay Kit (Promega, Shanghai, China), also known as MTS assay, according to the manufacturer’s instructions. BioTek ELx800 microplate reader (Bio‐Tek Instruments, Winooski, VT, USA) was used to detect the absorbance of each well at 490 nm.

For evaluation of drug sensitivity, an initial density of 10 000 cell per well was adopted to reseed the cells into 96‐well plates (Zhang *et al.*, 2016). The cells were incubated for 24 h until adherence. The medium was replaced by freshly prepared medium containing cisplatin and EHop‐016, which were both added at the indicated concentrations. The cells were cultivated for 24 h and treated with the MTS method. The IC_50_ and inhibition ratio were calculated using graphpad prism 7 software (Graphpad Prism Software Inc., San Diego, CA, USA).

### 2.7 EdU (5‐ethynyl‐2′‐deoxyuridine) incorporation assay

Cell proliferation assays were implemented using BeyoClick™ EdU Cell Proliferation Kit with Alexa Fluor 488 (Beyotime Biotechnology, Haimen, China) according to the manufacturer’s instructions. After receiving treatments identical to those in MTS assays, KYSE150 and KYSE510 cells were incubated with 10 µm EdU for 2 h at 37 °C. The cells were then proceeded to the fixation step with 3% paraformaldehyde in PBS and to the permeabilization step with 0.5% Triton X‐100 at room temperature. The fixatives were removed and the cells were washed by 3% BSA in PBS. Subsequently, ESCC cells were incubated and protected from light in Click Additive Solution and stained with DAPI. The fluorescence images of EdU incorporation samples were then obtained under ZEISS Axio Observer A1 (Carl Zeiss, Oberkochen, Germany) and photographed. The cells were further analyzed by calculating the ratio of EdU incorporation cells to the total number of the cells.

### 2.8 Wound healing assay

Wound healing assays were performed as described in our previous research (Zhang *et al.*, 2008). Transfected cells were incubated in 6‐well plates until full confluence, and cultured using serum‐free medium for 12 h in order to achieve quiescence. The scratches were produced by sterile pipette tips. Cell washing by PBS was performed, and the cells were incubated in the medium supplemented with 2% FBS.

To make sure that the measurements were taken at the same locations, calibrated scale on the IX73 inverted microscope (Olympus, Tokyo, Japan) was used to record the locations. Micrographs of the assigned areas were taken after 0, 24, and 48 h of incubations. The areas of wound healing were analyzed from 6 images, using imagej software (US National Institutes of Health, Bethesda, MD, USA).

### 2.9 Transwell assay

Cells were treated with serum starvation for 12 h (Li *et al.*, 2017) and further resuspended in serum‐free medium and reseeded onto the Falcon Chambers (BD Biosciences, Franklin Lakes, NJ, USA) with a density of 1 × 10^5^ cells per well. After 48 h, the cells that migrated toward the lower chambers were stained with 0.5% crystal violet. Each assay was photographed for 9 views under the IX73 inverted microscope (Olympus), and the number of cells within each chamber was counted by imagej software.

### 2.10 RAC1 activity assay

RAC1 activity was evaluated using Rac1 Pull‐down Activation Assay Biochem Kit (Cytoskeleton). In brief, cell lysis buffer was used to lyse the cells under different treatments (at 24 h after treatment with drug or 48 h after RAC1 transfection), and western blot was performed for quantification of total RAC1 using the collected lysates. The mixture of the sample and 20 μg GST‐PAK PBD beads, which bind to the active GTP‐RAC1 form, was rotated at 4 °C for 1 h. Beads were washed by wash buffer and resuspended by loading buffer. Proteins were separated on 10% SDS/PAGE and transferred to PVDF membranes for western blot. The total and activated RAC1 were detected by western blot using an anti‐RAC1 monoclonal antibody as described by the manufacturer.

### 2.11 Flow cytometry assay

Cell cycle and apoptosis were evaluated by the propidium iodide (PI) staining and the Annexin V–PI double staining assay. Cells were incubated with different drugs for 24 h and washed by ice‐cold PBS twice. After centrifugation, cells were resuspended and stained with PI or Annexin V–PI using the Cell Cycle Detection Kit (Beyotime Biotechnology) or the Annexin V‐FITC/PI Apoptosis Detection kit (Beyotime Biotechnology) according to the manufacturer's protocols. The flow cytometry data for cell cycle and apoptosis were analyzed by the modfit (Verity Software House, Topsham, ME, USA) and flowjo (TreeStar, Ashland, OR, USA) software. The synergistic effect of the combination of cisplatin and EHop‐016 was calculated using the response additivity approach (Foucquier and Guedj, 2015; Slinker, 1998).

### 2.12 RNA‐sequencing analysis and identification of differentially expressed mRNAs

Total RNA was extracted using the TRIzol (Invitrogen) method 24 h after cells were treated with drugs. Further library construction and Illumina’s HiSeq 2000 technology sequencing were performed by Novogene (Beijing, China). Data were extracted using tophat (v2.0.6; Tophat, Washington, MD, USA), and differentially expressed mRNAs were identified by deseq version 1.14.0 (Anders and Huber, 2010). A fold change cutoff of log_2_ < −1.5 or > 0.5 and a *P*‐value cutoff of *P* < 0.05 were adopted to select differentially expressed and significantly regulated gene sets. Functional enrichment by Gene Ontology (GO) and Kyoto Encyclopedia of Genes and Genomes (KEGG) analysis was performed to infer potential biological processes and pathways of methylation‐associated genes through the david Bioinformatics Tool (version 6.8; Ashburner *et al.*, 2000; Huang *et al.*, 2009). Results with a *P*‐value < 0.05 were considered as significant functional categories.

### 2.13 Quantitative reverse transcription polymerase chain reaction

Total RNA was extracted by the TRIzol (Invitrogen) method. The cDNAs were generated from reverse transcription using PrimeScript™ RT‐PCR kit (TaKaRa). Reverse transcription and quantitative reverse transcription polymerase chain reaction (qRT‐PCR) were conducted as described previously (Wu *et al.*, 2013). qRT‐PCR assays were performed on ABI 7500 Real‐Time PCR system with the primers (Table S3) using SYBR Premix Ex Taq (TaKaRa) under the manufacturer's instructions. The relative expression values of RAC1 were calculated by the ^ΔΔ^ *C*_t_ method and normalized to β‐actin in each sample.

### 2.14 Cellular metabolism assays

Alterations of cellular metabolism about glycolysis were measured using Glucose‐Glo™ Assay, Lactate‐Glo™ Assay, CellTiter‐Glo^®^ Luminescent Cell Viability Assay, and Glucose Uptake‐Glo™ Assay (Promega, Madison, WI, USA).

To detect the effects of RAC1 silencing and overexpression, KYSE150 and KYSE510 cells were seeded in 6‐well plates and transfected with siRAC1, siNC, empty vector, or RAC1 plasmid. After transfection, these cells were transplanted in 96‐well plates. To investigate the effectiveness of EHop‐016 in overcoming cisplatin resistance, ESCC cells were seeded in 96‐well opaque luminescent plates, and then, cisplatin and EHop‐016 were introduced according to the MTS assay. After proper cultivation, the culture medium and the original medium (baseline control) were completely removed and collected. Glucose (Glucose‐Glo™ Assay) and lactate (Lactate‐Glo™ Assay) levels were quantified using the medium according to the manufacturer’s instructions. After removal of the medium, the remaining cells were washed by PBS twice and harvested. Cellular ATP production levels were measured using the firefly luciferase method (CellTiter‐Glo^®^ Luminescent Cell Viability Assay). The bioluminescent glucose uptake assay (Glucose Uptake‐Glo™ Assay) was also conducted using the standard protocol provided by the manufacturer. Luminescence of all of the above metabolism assays was determined by the luminometer (Promega GloMax^®^ 96 Microplate Luminometer). All experiments were performed in triplicate and repeated three times.

### 2.15 Xenograft studies

This study was approved by the Ethics Committee of Shantou University Medical College, and all mice were treated humanely. Twenty‐eight female *nu/nu* mice (Vital River Laboratories Animal Technology, Beijing, China) at 6–8 weeks of age were housed in a specific pathogen‐free environment and allowed for adaption to their environment before experiments. For cell inoculation, KYSE150 cells (2 × 10^6^ cells·mL^−1^) were injected subcutaneously into the armpit of the mice. Drug injection began when the average tumor volume reached 100 mm^3^. Cisplatin (2 mg·kg^−1^ body weight; Selleck), EHop‐016 (20 mg·kg^−1^ body weight; MedChem Express), or combination therapy (cisplatin plus EHop‐016) was administered by intraperitoneal injection every 3 days. Animals were monitored every 3 days, and tumor volume was determined by the formula (width^2^ × length)/2 using a slide caliper. Twenty‐seven days after inoculation of tumor cells, the mice were euthanized with an overdose of diethyl ether, and the tumors were resected surgically. Examination of the tumor cells was conducted by hematoxylin and eosin (H&E) staining and IHC detection.

### 2.16 Statistical analysis

Experiments were performed in triplicate and repeated three times. Statistics obtained from each assay were imported into graphpad prism 7 (Graphpad Prism Software Inc., San Diego, CA, USA) and spss 17.0 software (SPSS Inc., Chicago, IL, USA) for graphing and analysis. All experimental results are presented as mean ± SD. Statistical differences between samples were analyzed using Student’s* t*‐tests for independent samples. *P*‐value < 0.05 was defined as statically significant.

## 3 Results

### 3.1 High RAC1 expression is related to poor prognosis in patients with ESCC

**Figure 1:**
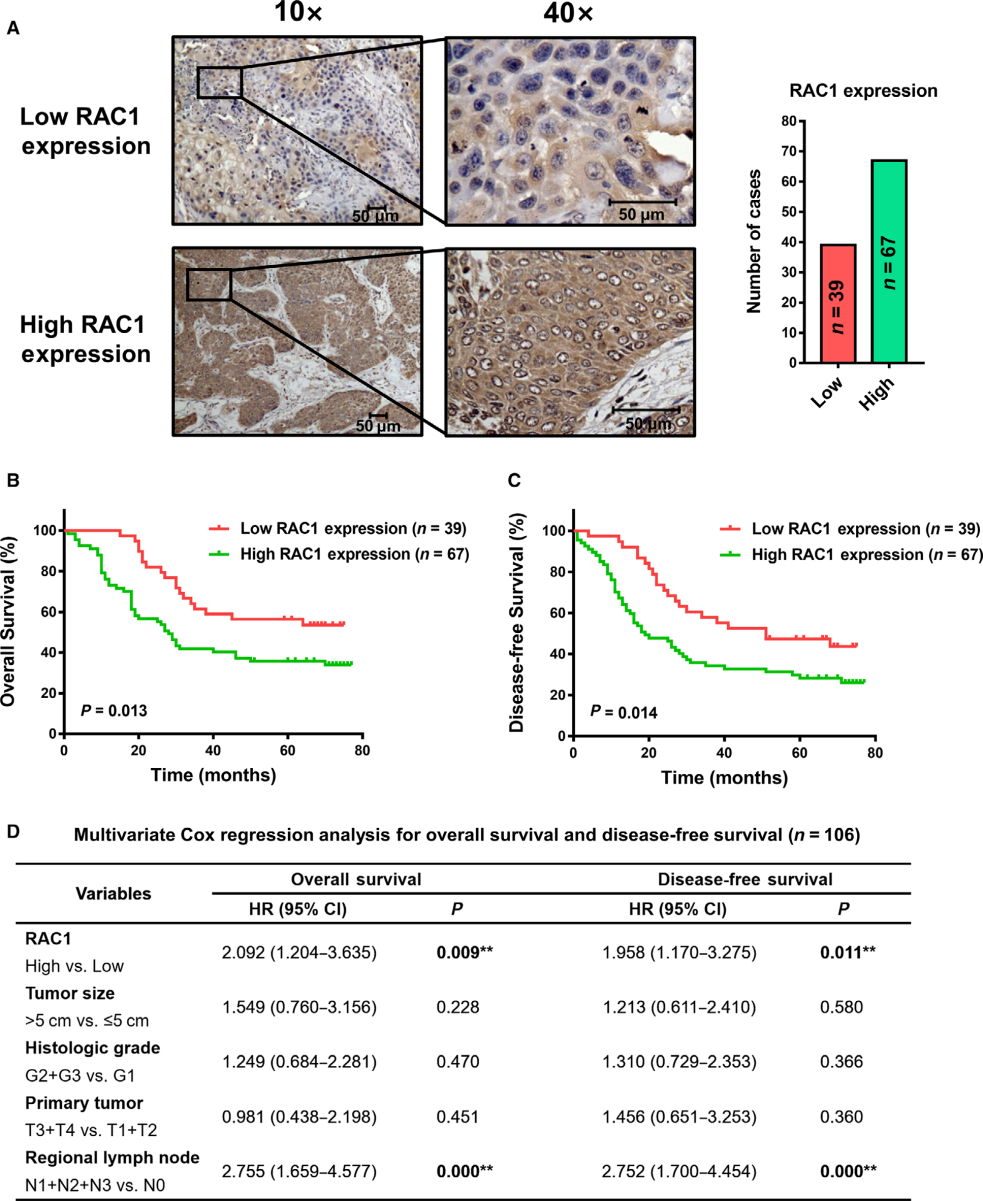
IHC staining of RAC1 and prognostic significance evaluation in ESCC patient samples. (A) IHC detection of RAC1 expression in 106 ESCC patient samples. (B, C) Kaplan–Meier survival analysis with log‐rank test evaluating the OS and DFS in patients expressing high or low levels of RAC1. (D) Multivariate Cox regression models for OS and DFS were performed. All scale bars, 50 μm.

We evaluated RAC1 expression in 106 tumor samples of ESCC patients using IHC and anti‐RAC1 monoclonal antibody. As illustrated in Fig. 1A, 39 (36.8%) tumors expressed low RAC1 levels, whereas 67 (63.2%) tumors expressed high levels of RAC1. Poorer overall survival (OS; *P* = 0.013; Fig. 1B) and disease‐free survival (DFS; *P* = 0.014; Fig. 1C) in the high‐RAC1‐expression group were revealed by Kaplan–Meier analysis. Additionally, as illustrated in Table S2, higher RAC1 expression in tumors was significantly associated with larger tumor sizes (*P* < 0.05), lymph node metastasis (*P* = 0.001), and poorer clinical stages (*P* < 0.001). Analysis by multiple Cox regression analysis illustrated that expression of RAC1 was an independent factor for both OS [HR = 2.092, 95% confidence interval (CI) = 1.204–3.635, *P* < 0.01] and DFS (HR = 1.958, 95% CI = 1.170–3.275, *P* = 0.011; Fig. 1D).

### 3.2 RAC1 promotes proliferation and migration of ESCC cells

**Figure 2:**
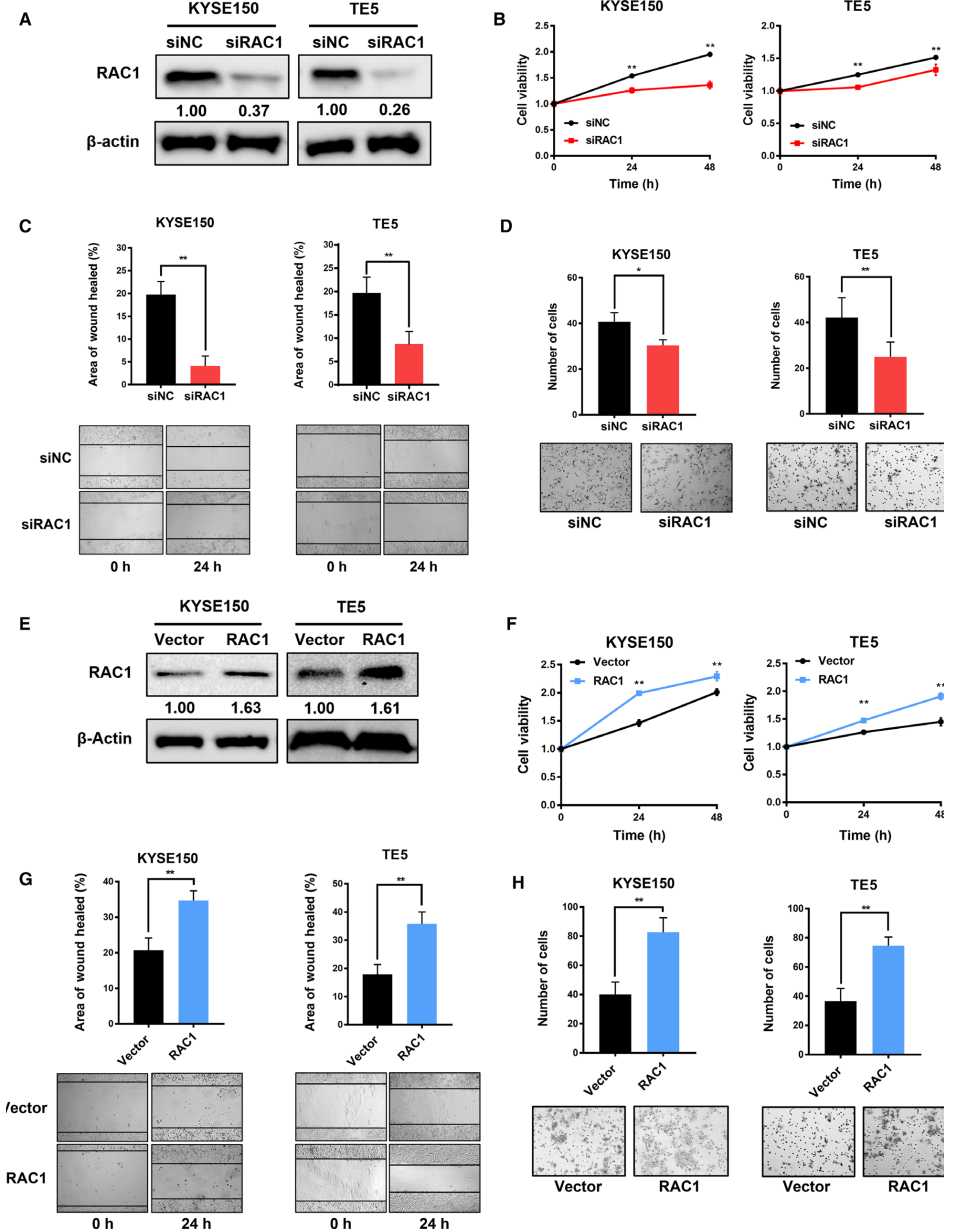
Effects of RAC1 on the proliferation and migration of ESCC cells. (A) Western blot analysis of RAC1 expression in both KYSE150 and TE5 cells at 48 h after transfection with siNC (control siRNA) or siRAC1. (B) MTS assay was performed at 24 and 48 h after siRNA transfection. (C) Representative images and quantitative analysis of the results from the wound healing assay. (D) Migration rate was measured using Transwell assay. (E) RAC1 expression in ESCC cells transfected with the empty vectors (control) or RAC1 plasmids was measured by western blot at 48 h after transfection. (F) MTS assay was performed at 24 and 48 h after the transfection of empty vectors and RAC1 plasmids. (G) Migration rate was measured using wound healing assay. (H) Representative images and quantitative analysis of the results from the Transwell assay. *P < 0.05; **P < 0.01. Statistical differences were analyzed using Student’s* t*‐tests. Error bars represent SD from triplicate experiments.

To identify the role of RAC1 in development and progression of ESCC cells, we set up a RAC1 downregulation model by siRNA transfection, which was examined by western blot at 48 h after transfection (Fig. 2A). Next, we performed MTS, EdU, wound healing, and Transwell assays to explore the role of RAC1 in tumor proliferation and migration. When RAC1 was downregulated by siRAC1, the proliferation ability was decreased in both KYSE150 and TE5 cells (Fig. 2B, Fig. S1A). In the wound healing assays, the siRNA‐induced downregulation of RAC1 led to the decrease in wound healing rate, that is, decrease in the cell migration ability (Fig. 2C). In the Transwell assays, the decrease in RAC1 expression gave rise to a decrease in the number of cells invading through the chamber (Fig. 2D). In contrast to the RAC1 downregulation model, we set up an upregulation model through plasmid transfection, which was determined by western blot at 48 h after transfection (Fig. 2E). In the MTS and EdU assays, when RAC1 was upregulated by RAC1 plasmids, the proliferation ability of the cells was increased, compared to those cells transfected with empty vector (Fig. 2F, Fig. S1B). In the wound healing assays, the upregulation of RAC1 resulted in the increase in wound healing rate (Fig. 2G). In the Transwell assays, the increase in RAC1 expression resulted in the increase in cell migration ability (Fig. 2H). Taken together, the data indicated that RAC1 plays a tumor‐promoting role in ESCC cells.

### 3.3 RAC1 confers cisplatin resistance to ESCC *in vitro*

**Figure 3:**
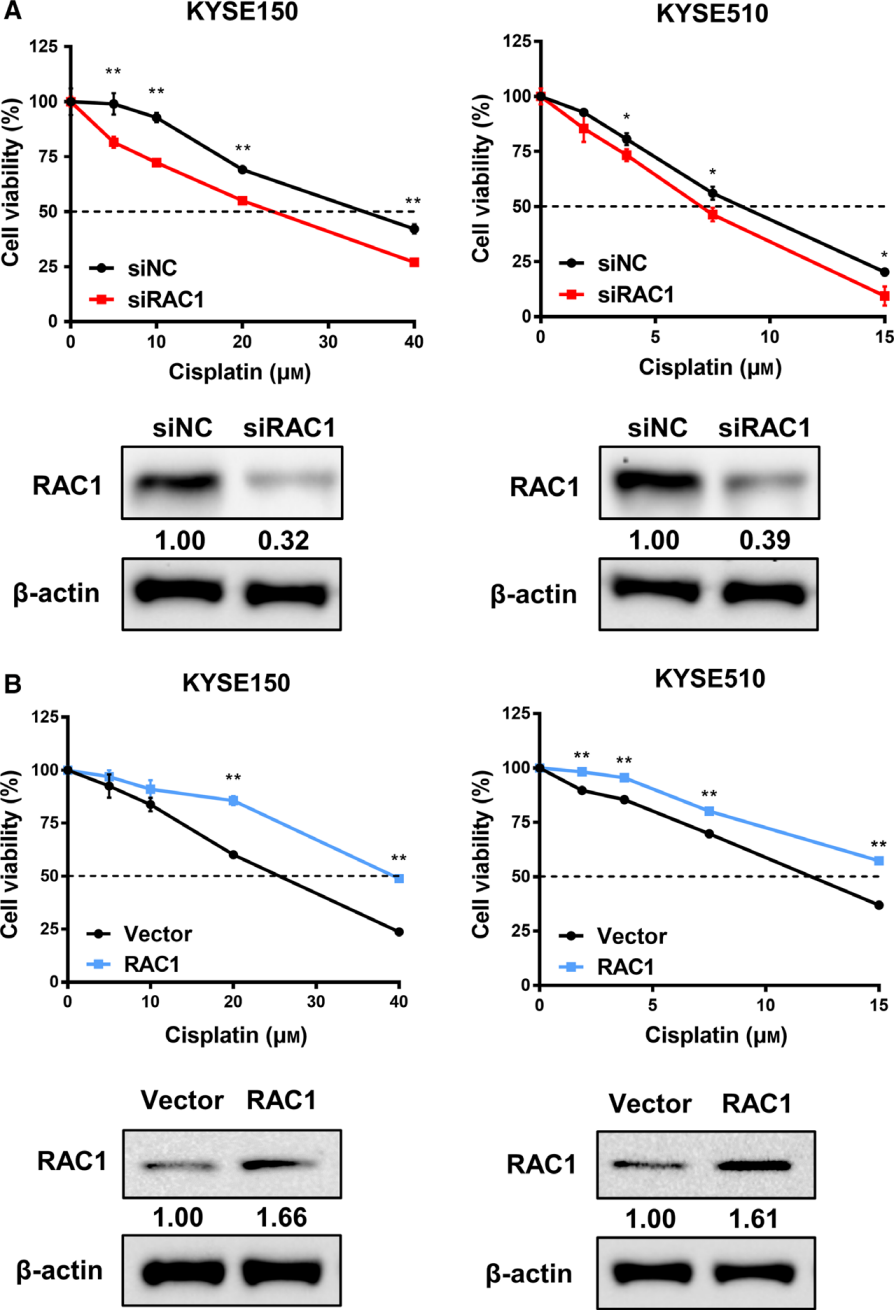
Correlation between RAC1 and cisplatin resistance in ESCC cells. (A) Chemoresistance was measured using MTS assay, and expression of protein in the siRNA‐transfected KYSE150 and KYSE510 cells was evaluated by western blot. (B) Chemoresistance was determined using MTS assay, and protein level was detected by western blot in the KYSE150 and KYSE510 cells transfected with empty vectors or RAC1 plasmids. *P < 0.05; **P < 0.01. Statistical differences were analyzed using Student’s* t*‐tests. Error bars represent SD from triplicate experiments.

Chemoresistance remains the leading cause of treatment failure in clinical practice (Perez *et al.*, 1990; Riganti *et al.*, 2015), and no study has revealed the relationship between RAC1 and chemoresistance of ESCC. We therefore evaluated the impact of RAC1 on chemoresistance of ESCC cells, using the gold‐standard chemotherapeutic drug cisplatin. The expression of RAC1 under knockdown or overexpression was examined using western blot at 48 h after transfection as shown in Fig. 3A,B. We analyzed the response of ESCC cells to cisplatin after RAC1 knockdown or overexpression using cell viability assay. The cisplatin resistance of KYSE150 or KYSE510 cells transfected with siRAC1 was markedly decreased than that of the cells transfected with siNC (Fig. 3A, Fig. S1C). In contrast, the cisplatin resistance of RAC1‐plasmid‐transfected cells was markedly increased than that of the empty‐vector‐transfected cells (Fig. 3B, Fig. S1D).

### 3.4 Combination therapy of cisplatin and RAC1 inhibitor reverses the chemoresistance to cisplatin *in vitro*

**Figure 4:**
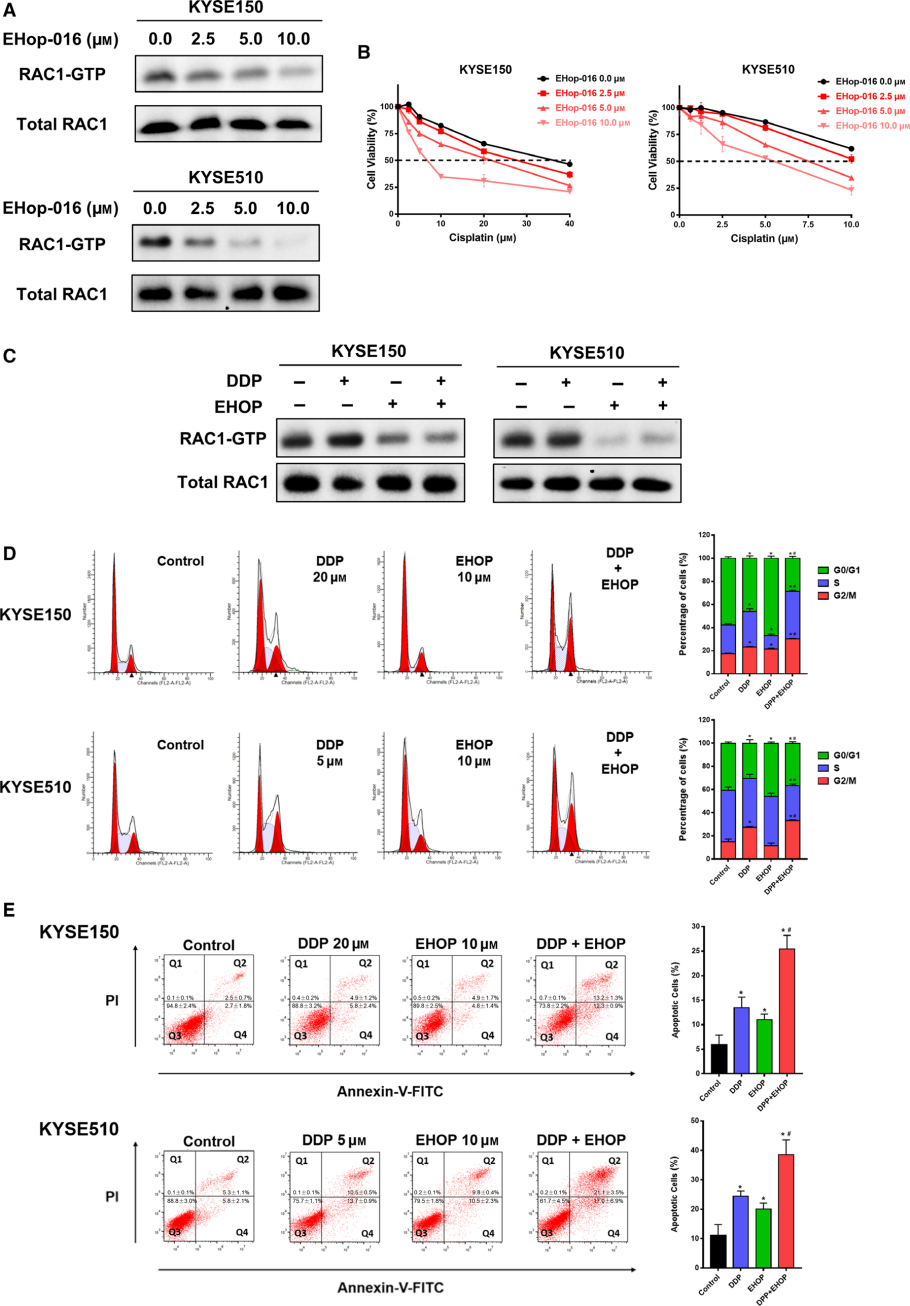
Antitumor effects of combination therapy of cisplatin and RAC1 inhibitor in ESCC cells. (A) RAC1 activity in KYSE150 and KYSE510 cells treated with 0.0, 2.5, 5.0, and 10.0 μm RAC1 inhibitor EHop‐016 was measured by pull‐down assay for RAC1‐GTP. Representative western blot images demonstrate positive bands for RAC1‐GTP and total RAC1. (B) Cell viability was evaluated at 48 h after cisplatin and EHop‐016 treatment. (C) RAC1 activity in KYSE150 and KYSE510 cells after treatment with the indicated concentration of cisplatin (DDP), EHop‐016 (EHOP), or combination therapy (DDP and EHOP) was determined by pull‐down assay and western blot. (D) Cell cycle analysis using flow cytometry in KYSE150 and KYSE510 cells treated with the indicated concentration of cisplatin, EHop‐016, or combination therapy for 24 h. (E) The apoptotic rates of KYSE150 and KYSE510 cells treated with the indicated concentration of cisplatin, EHop‐016, or combination therapy for 24 h were measured by flow cytometry. *P < 0.05 vs. control; ^#^ *P* < 0.05 vs. cisplatin. Student’s* t*‐tests were used to analyze statistical differences. Error bars represent SD from triplicate experiments.

Due to the promoting role of RAC1 in cisplatin resistance of ESCC cells, we hypothesized that RAC1 inhibitor could overcome cisplatin resistance. The chosen concentrations of EHop‐016 were derived from our pre‐experiment that examined the effect of EHop‐016 treatment alone on the cell viability of different ESCC cell lines (Fig. S2). Figure 4A demonstrates the inhibition of RAC1 activity under different concentrations of EHop‐016. Then, we used both cisplatin and RAC1 inhibitor EHop‐016 to treat ESCC cells and observed the survival rate of cells using cell viability assay. As shown in Fig. 4B and Fig. S1E, the cisplatin resistance of KYSE150 and KYSE510 cells was decreased proportionally to the concentration of EHop‐016 when compared to the control group (0 μm EHop‐016).

### 3.5 Combination therapy of cisplatin and RAC1 inhibitor enhances cisplatin‐induced G2/M phase cycle arrest and apoptosis *in vitro*

The effectiveness of combination therapy to cisplatin‐induced cell cycle arrest and apoptosis was further investigated. Cells were treated with either cisplatin monotherapy (KYSE150: 20 μm; KYSE510: 5 μm), EHop‐016 monotherapy (KYSE150, KYSE510: 10 μm), or combination therapy (cisplatin and EHop‐016) for 24 h, followed by PI staining or Annexin V–PI double staining for flow cytometry. Figure 4C shows the changes in RAC1 activity under different treatments. As shown in Fig. 4D, the combination therapy induced a strong cell cycle arrest effect. Compared to monotherapy of cisplatin, the G2/M cell cycle arrest rate was significantly elevated (KYSE150: 30.3% vs. 23.14%; KYSE510: 33.13% vs. 27.32%). As demonstrated in Fig. 4E, when cisplatin was combined with EHop‐016, the cell apoptotic rate was significantly increased, compared to monotherapy of cisplatin (KYSE150: 25.5% vs. 13.46%; KYSE510: 38.5% vs. 24.5%). After calculation through the response additivity approach, the combination therapy showed a synergistic effect in inducing cell apoptosis (data not shown).

### 3.6 RAC1 inhibition suppresses glycolysis in ESCC cells

**Figure 5:**
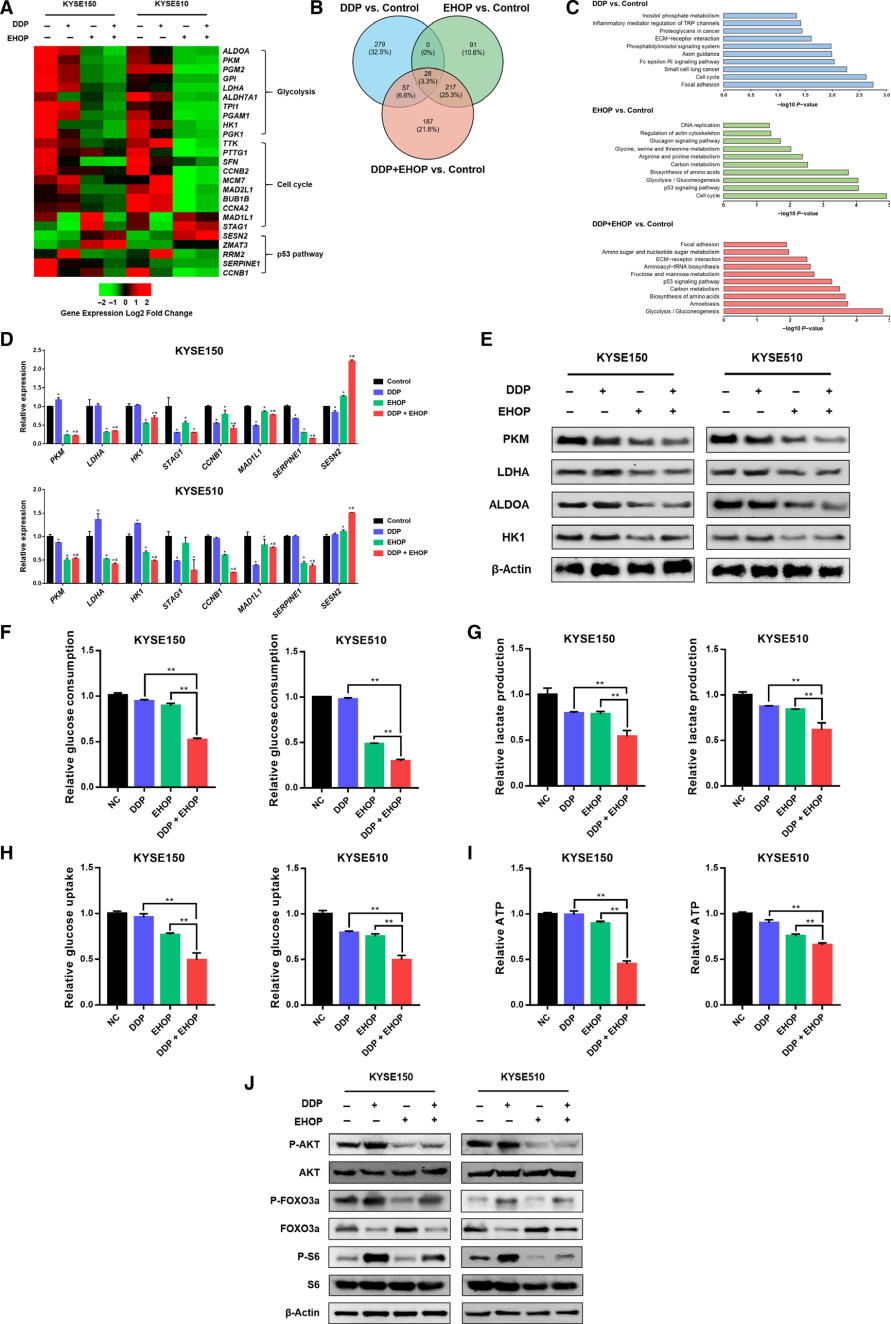
Inhibition of RAC1 dampens glycolysis via inhibition of AKT signaling. (A) RNA‐seq analysis represented by a heatmap of gene expression for glycolysis, cell cycle and p53 pathway in KYSE150 and KYSE510 cells after treatment with cisplatin (DDP), EHop‐016 (EHOP), or combination therapy (DDP and EHOP) (*P* < 0.001, and fold change > 1.5 log_2_). (B) A Venn diagram demonstrates the number of differentially expressed mRNAs for cisplatin monotherapy, EHOP‐016 monotherapy, or combination therapy versus control. (C) Differentially expressed mRNAs in each treatment group versus control were categorized using GO enrichment analysis. (D) Validation of RNA‐seq data of selected genes by qRT‐PCR (*P < 0.05 vs. control; ^#^ *P* < 0.05 vs. cisplatin). (E) The expression of glycolytic enzymes PKM, LDHA, ALDOA, and HK1 was examined using western blot. (F) Glucose consumption in KYSE150 and KYSE510 cells after being treated with cisplatin, EHop‐016, or combination therapy. (G) Lactate production of KYSE150 and KYSE510 cells was measured under the treatment of cisplatin, EHop‐016, or combination therapy. (H) Glucose uptake was determined after KYSE150 and KYSE510 cells were treated with cisplatin, EHop‐016, or combination therapy. (I) ATP production in KYSE150 and KYSE510 cells that were treated with cisplatin, EHop‐016, or combination therapy. *P < 0.05; **P < 0.01. (J) Western blot analysis of phospho‐AKT (P‐AKT), AKT, phospho‐FOXO3a (P‐FOXO3a), FOXO3a, phospho‐S6 (P‐S6), and S6. Statistical differences were analyzed using Student’s* t*‐tests. Error bars represent SD from triplicate experiments.

To explore the molecular mechanisms by which RAC1 inhibitor reverses chemoresistance of ESCC cells to cisplatin, RNA sequencing (RNA‐seq) (SRP173519) was used to compare mRNA profiles among each treatment group. The heatmap demonstrated that after being treated with RAC1 inhibitor or combination therapy, the glycolysis, cell cycle, and p53 pathways in both KYSE150 and KYSE510 cells were significantly inhibited, when compared to the control group or cisplatin monotherapy (Fig. 5A). A large number of genes were significantly up‐ or downregulated under different treatments (Fig. 5B). GO and DAVID enrichment analysis yielded the top 10 most significantly changed terms as demonstrated in Fig. 5C. Importantly, the GO term glycolysis/gluconeogenesis was the third and the most significantly changed term in the RAC1 inhibitor monotherapy group and the combination therapy group, respectively (Fig. 5C).

For further validation, the assays for detecting the effect of RAC1 inhibition on glycolysis were performed subsequently on the RNA level, the protein level, and finally the cellular metabolism level. First of all, as illustrated in Fig. 5D, qRT‐PCR validation for eight selected differentially expressed mRNAs was consistent with the RNA‐seq results (Fig. 5A). Next, we determined whether the same results were observed on the protein level. The enzymes critical for aerobic glycolysis, including PKM, LDHA, ALDOA, and HK1, were suppressed under treatment with RAC1 inhibitor or combination therapy (Fig. 5E). Finally, we determined whether the effect of combination therapy on RAC1 inhibition eventually altered the cellular metabolism. In our study, we mainly focus on the effect of RAC1 inhibition on reversing the cisplatin resistance in ESCC cells from the aspect of glycolysis. As illustrated in Fig. 5F–I, with the combination of cisplatin and EHop‐016, significant decreases in glucose consumption (Fig. 5F), lactate (Fig. 5G) and ATP production (Fig. 5I), and glucose uptake (Fig. 5H) were observed in both the KYSE150 and KYSE510 cells, when compared to the cisplatin or EHop‐016 single‐treatment groups (*P* < 0.001). Based on these results, inhibition of RAC1 overcomes the cisplatin resistance and suppresses ESCC cell glycolysis.

**Figure 6:**
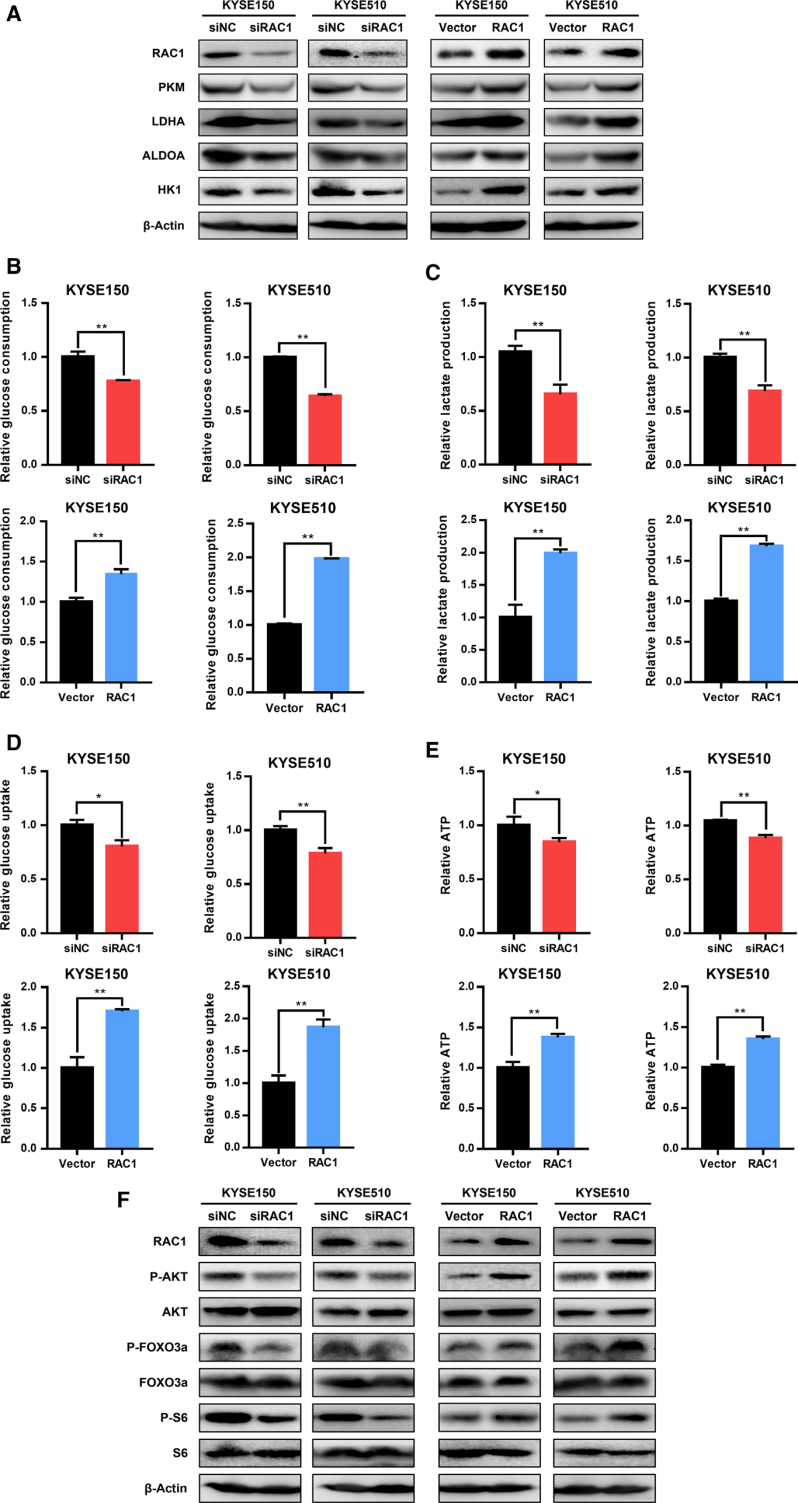
Effects of RAC1 on glycolysis in ESCC cells. (A) Western blot analysis of glycolytic enzymes PKM, LDHA, ALDOA, and HK1 under RAC1 silencing or overexpression. (B) Glucose consumption of KYSE150 and KYSE510 cells that were transfected with siRAC1 or RAC1 plasmid. (C) Lactate production in KYSE150 and KYSE510 cells transfected with siRAC1 or RAC1 plasmid was measured. (D) Glucose uptake was detected in KYSE150 and KYSE510 cells after transfection with siRAC1 or RAC1 plasmid. (E) ATP generation in KYSE150 and KYSE510 cells was determined under transfection of siRAC1 and RAC1 plasmid. *P < 0.05; **P < 0.01. (F) Western blot analysis of phospho‐AKT (P‐AKT), AKT, phospho‐FOXO3a (P‐FOXO3a), FOXO3a, phospho‐S6 (P‐S6) and S6 under RAC1 silencing or overexpression. Student’s* t*‐tests were used to analyze statistical differences. Error bars represent SD from triplicate experiments.

To further verify the relationship between RAC1 suppression/overexpression and glycolysis, RAC1 was directly knocked down or overexpressed. The consequences on glycolysis corroborated our proposed mechanism. The critical enzymes for aerobic glycolysis, including PKM, LDHA, ALDOA, and HK1, were suppressed under RAC1 silencing but upregulated in RAC1 overexpression as shown Fig. 6A. Similarly, the alterations of cellular metabolism were detected under RAC1 silencing or overexpression. According to our results on glucose consumption (Fig. 6B), lactate production (Fig. 6C), glucose uptake (Fig. 6D), and ATP production (Fig. 6E), significant reduction of glycolysis under RAC1 silencing and increases under RAC1 overexpression were observed in both KYSE150 and KYSE510 cells. Taken together, these results are highly consistent with our proposed mechanism that RAC1 inhibition suppresses glycolysis in ESCC cells.

### 3.7 Inhibition of RAC1 suppresses glycolysis via blocking AKT/FOXO3a signaling in ESCC cells

It is well known that AKT exerts a direct influence on glucose metabolism (Robey and Hay, 2009). It is also reported that there exists a mutual regulation of RAC1 and AKT, which shows that RAC1 acts as an upstream regulator of AKT and vice versa (Kwon *et al.*, 2000; Murga *et al.*, 2002; Zhu *et al.*, 2015). Knowing that RAC1 deletion suppresses the activation of downstream targets of AKT (Saci *et al.*, 2011), therefore, we hypothesized that the suppressive effect of RAC1 inhibitor on the glycolytic enzymes was due to the inhibition of AKT pathway. As shown in Fig. 5J, phosphorylation of AKT was drastically suppressed by RAC1 inhibitor treatment or combination therapy. Interestingly, treatment with cisplatin significantly enhanced the phosphorylation of AKT, while the expression of AKT remained unchanged (Fig. 5J). Furthermore, we investigated the downstream proteins of AKT pathway such as FOXO3a and S6, which are also known to involve in regulation of glycolysis (Khatri *et al.*, 2010). Their activation also tightly depended on the mammalian target of rapamycin (mTOR) activation, which indicated that mTOR can be critical in regulating cancer metabolism on the downstream of RAC1 (Esen *et al.*, 2013). Our findings demonstrated that the phosphorylation of FOXO3a and S6, which served as the markers of mTOR activity (Gödel *et al.*, 2011; Saci *et al.*, 2011; Sarbassov *et al.*, 2005), was drastically increased under cisplatin treatment and decreased under RAC1 inhibitor or combination therapy, when compared to the control group or the cisplatin monotherapy group (Fig. 5J). The levels of FOXO3a were inversely correlated to the levels of P‐FOXO3a, while S6 levels were not altered under different treatments (Fig. 5J). Consistently, the phosphorylation of AKT, FOXO3a, and S6 was also decreased with RAC1 downregulation and increased under RAC1 overexpression (Fig. 6F).

In order to validate the role of FOXO3a in regulating glycolytic enzymes, the effects of FOXO3a knockdown on the glycolytic enzymes were detected by western blot. As shown in Fig. S3, FOXO3a silencing decreased the expression of P‐FOXO3a, PKM, LDHA, ALDOA, and HK1, which indicated that FOXO3a silencing had an inhibitory effect on the glycolytic enzymes. In summary, inhibition of RAC1 suppresses glycolysis through the AKT/FOXO3a pathway.

### 3.8 Combining chemotherapy with RAC1 inhibitor results in enhanced antitumor effects by suppressing the glycolytic enzymes in ESCC xenograft mice

**Figure 7:**
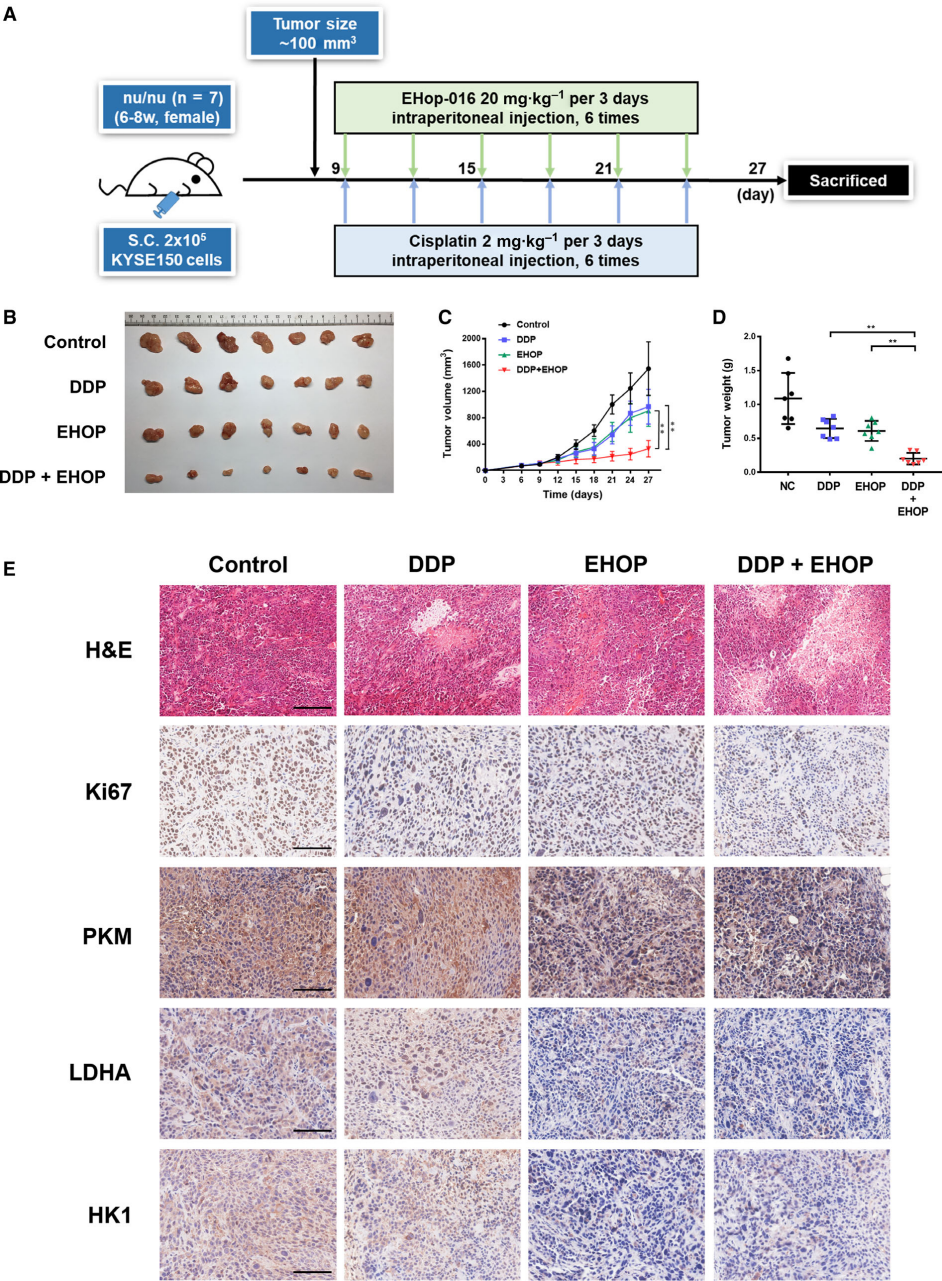
Combining chemotherapy with RAC1 inhibitor enhances therapeutic effects in ESCC xenograft mouse models. (A) Summary of *in vivo* study: Female *nu/nu* mice (6–8 weeks of age, *n* = 28) were inoculated with 2 × 10^5^ KYSE150 cells (2 × 10^6^ cells·mL^−1^, 100 μL per mouse). The mice received intraperitoneal injection of cisplatin (DDP), EHop‐016 (EHOP), or combination of drugs (DDP and EHOP) every 3 days when tumor volumes reached approximately 100 mm^3^. The mice were euthanized 27 days after the inoculation of cells. (B) Tumors were resected at day 27. (C) Tumor volumes were evaluated every 3 days. (D) Tumor weights were determined at day 27. (E) Representative images for H&E staining and immunohistochemical analysis of Ki67, PKM, LDHA, and HK1 in different treatment groups. All scale bars, 100 μm. *P < 0.05; **P < 0.01. Statistical differences were analyzed using Student’s* t*‐tests. Error bars represent SD.

To assess the *in vivo* therapeutic effects of combination therapy of cisplatin and EHop‐016, we established an ESCC xenograft tumor model by implanting KYSE150 cells subcutaneously in *nu/nu* mice (females, 6–8 weeks of age). Cisplatin and EHop‐016 were injected intraperitoneally every 3 days for six times after the tumor volume reached approximately 100 mm^3^ (Fig. 7A). The combination of cisplatin and EHop‐016 significantly suppressed tumor volume and tumor weight, compared to the control group or each monotherapy (Fig. 7B–D). The tumor necrotic area was significantly increased, while the expression of Ki67 was significantly reduced in tumors of combination therapy group, compared to that of control group or each monotherapy (Fig. 7E). More importantly, the expression of enzymes for glycolysis, including PKM, LDHA, and HK1, was significantly suppressed under the injection of RAC1 inhibitor (Fig. 7E).

## 4 Discussions

Accumulating evidence has shown that RAC1 is involved in the development and progression of tumors (Myant *et al.*, 2013; Wang *et al.*, 2015). However, the role of RAC1 remains controversial, indicating that RAC1 is involved in a complex network of regulation in tumors. For example, although RAC1 is generally thought to play a tumor‐promoting role in tumor development (Baugher *et al.*, 2005; Chen *et al.*, 2011; Frances *et al.*, 2015), some research indicates that RAC1 suppresses tumor progression in renal carcinomas and skin cancers (Engers *et al.*, 2001; Malliri *et al.*, 2002). Our results indicate that high RAC1 levels in tumor correlate with poor prognosis in ESCC patients, and it is confirmed by our *in vitro* data, which demonstrate that RAC1 positively regulates proliferation and migration of ESCC cells. Thus, RAC1 plays a tumor‐promoting role in the progression and development of ESCC. In clinical practice, RAC1 possesses great potentials to be a biomarker to evaluate the prognosis of ESCC patients.

Clinically, cisplatin is one of the most effective chemotherapeutic drugs to treat various malignancies, such as testicular, ovarian, and esophageal cancers (Eljack *et al.*, 2014). Despite treatment advances, tumor resistance to cisplatin remains the major cause of treatment failure (Perez *et al.*, 1990; Riganti *et al.*, 2015). Previous studies indicate that the involvement of RAC1 in regulating chemoresistance differs in various types of cancers. For example, in lung cancer and head and neck cell carcinoma, silencing of RAC1 is related to higher cisplatin sensitivity (Chen *et al.*, 2011; Skvortsov *et al.*, 2014). In contrast, in epidermoid carcinoma or liver carcinoma, downregulation of RAC1 results in a decrease in cisplatin sensitivity (Shen *et al.*, 2004). However, to the best of our knowledge, there are two major limitations for the current studies about RAC1 and chemotherapy. First, no *in vivo* study for combination therapy using RAC1 inhibitor and chemotherapeutic drugs has been conducted. Second, the molecular mechanisms by which RAC1 regulates chemoresistance are largely unknown. Our study unveils that the expression of RAC1 is positively related to cisplatin resistance. Thus, inhibiting RAC1 can suppress both the development and chemoresistance of ESCC cells, and it is reasonable to use a RAC1 inhibitor for combination with chemotherapy. In comparison with RAC1 siRNA, a RAC1 inhibitor can produce a more potent effect, because siRNA does not completely eliminate the expression of RAC1 proteins (Gastonguay *et al.*, 2012). NSC23766 is the first developed and most widely used RAC1 inhibitor targeting the RAC1‐GEF interaction (Dütting *et al.*, 2015; Gao *et al.*, 2004; Levay *et al.*, 2013). However, the low potency (IC_50_ > 75 μm) of NSC23766 limits its use as a therapeutic agent (Gao *et al.*, 2004). Similarly, other RAC inhibitors, including AZA1, EHT 1864, IA‐116, and ZINC69391, also have high effective concentrations (IC_50_ = 10–50 μm) (Cardama *et al.*, 2014; Ferri *et al.*, 2009; Montalvo‐Ortiz *et al.*, 2012; Zins *et al.*, 2013). A recently synthesized RAC1 inhibitor, EHop‐016, blocks the GEF‐RAC interaction and is specific for RAC1 at concentrations lower than its IC_50_ (Humphries‐Bickley *et al.*, 2017; Montalvo‐Ortiz *et al.*, 2012). The potency of EHop‐016 is approximately 100 times higher than that of NSC23766 and 10–50 times higher than that of other RAC inhibitors; therefore, it holds the greatest potential as a targeted therapeutic, and for combination therapy with chemotherapeutic drugs both *in vitro* and *in vivo* (Bid *et al.*, 2013; Montalvo‐Ortiz *et al.*, 2012). Our data suggest that RAC1 inhibitor can reverse chemoresistance in both ESCC cells and xenograft mouse models.

Even under normal oxygen concentrations, cancer cells produce energy mainly via glycolysis in high rates, and this phenomenon (aerobic glycolysis) is a hallmark of cancer (Cerella *et al.*, 2014; Devic, 2016; Liberti and Locasale, 2016). Through overexpressing the corresponding key metabolic enzymes, this newly acquired metabolic profile is prone to decide multiple cancer hallmarks including resistance to cell demise (Cerella *et al.*, 2014). These findings indicate that glycolytic enzymes can also act as direct modulators of cell death, by relocalizing to subcellular compartments, including the nucleus, the plasma membrane, and the mitochondria, rather than function simply at the cytosolic levels as they were primarily expected to do (Sirover, 2012; Tristan *et al.*, 2011; Ucker *et al.*, 2012). In addition to its tumor‐promoting effects, aerobic glycolysis provides an environment that exacerbates drug resistance of cancer cells (Bhattacharya *et al.*, 2016). Hence, the enzymes that directly regulate glycolysis, especially hexokinase 1 (HK1), pyruvate kinase (PKM), lactate dehydrogenase A (LDHA), and aldolase A (ALDOA), are recruited in deteriorating chemoresistance (Li *et al.*, 2015c). HK1, which involves in the first step of most glucose metabolism pathways, is a rate‐limiting enzyme in glucose oxidation reaction (Li *et al.*, 2015c). PKM is responsible for catalyzing the last step of glycolysis and regarded as a rate‐limiting enzyme for glycolysis (Taniguchi *et al.*, 2015). LDHA and ALDOA are key enzymes for glycolysis and have been proved to promote drug resistance (Long *et al.*, 2014; Xie *et al.*, 2014). Our RNA‐seq results indicate that almost all the enzymes for glycolysis are suppressed under RAC1 inhibitor or combination therapy. Further validation by qRT‐PCR, western blot, and IHC for the selected glycolytic enzymes confirms the RNA‐seq results. Therefore, our study underlines the significance of RAC1 in regulating glycolysis.

Interestingly, the AKT signaling, which is considered to be the ‘glycolytic kinase’ that contributes to aerobic glycolysis (Robey and Hay, 2009), is shown to be significantly activated by cisplatin and can be suppressed by the RAC1 inhibitor in our study. As indicated in the current research, there exists a mutual regulation of RAC1 and AKT, which should be considered comprehensively. There are some studies clarifying that AKT acts as a direct (Zhu *et al.*, 2015) or indirect (Kwon *et al.*, 2000) upstream modulator of RAC1. In contrast, other studies indicate that RAC1 can function as an upstream regulator of AKT (Murga *et al.*, 2002). Our data indicate that under chemotherapy, ESCC cells utilize the activation of AKT signaling for adaptation and survival, while further administration of RAC1 inhibitor can reverse both the activation of AKT signaling and chemoresistance. These results are consistent with what we have interpreted previously that RAC1 is the upstream regulator of AKT in overcoming chemoresistance. In addition, over the past few years, drugs targeting AKT have been extensively developed and tested in clinical trials (Faes and Dormond, 2015). However, the negative feedback mechanisms have considerably decreased the potency of AKT inhibitors and caused undesired side effects (Chandarlapaty, 2012; Nitulescu *et al.*, 2016). Consequently, inhibiting the activation of RAC1, which is located upstream of AKT, can be an admissible strategy to maximize the therapeutic effects of AKT inhibitor in clinical practice.

There is an intriguing issue that the phosphorylated form of FOXO3a was slightly affected by the combination therapy with respect to DDP treatment alone. We suggest that because of the further downstream position of FOXO3a, RAC1 inhibition may cause fewer effects on FOXO3a expression under cisplatin‐induced FOXO3a activation. Apart from the AKT/FOXO3a pathway which is modulated by RAC1 as we proposed, it is also possible that there exists a more complicated regulatory mechanism, where some complementary pathways may be activated to counteract the effect of RAC1 on FOXO3a expression.

In addition, one of the approaches that RAC1 uses to control cancer cell metabolism is by interacting with mTOR. RAC1 has been proved to critically regulate both mTORC1 and mTORC2 by binding directly to them, localizing mTOR to specific membranes, and mediating their activation in response to growth factors (Esen *et al.*, 2013). RAC1 deletion inhibits the activation of the translational regulators 4eBP1 and p70 S6 kinase, which are the downstream targets of mTORC1 (Ma and Blenis, 2009), and suppresses the activation of AKT, which is controlled by mTORC2 (Saci *et al.*, 2011). To be more specific, mTORC1 is well known to modulate protein synthesis via phosphorylation of 4eBP1 and S6K1, the latter of which will further phosphorylate the ribosomal protein S6 (Ma and Blenis, 2009). mTORC2, which is recruited by RAC1, is reported to activate AKT by AKT Ser473 phosphorylation (Hresko and Mueckler, 2005; Jacinto et al., 2006; Saci *et al.*, 2011; Sarbassov *et al.*, 2005). In order to detect the mTOR activation, several representative markers are chosen for the study. Gödel et al. reported that pS6 can serve as a marker of mTORC1 activity (Gödel *et al.*, 2011), while the phosphorylation and activation of the kinases AKT represent the primary function of mTORC2 (Sarbassov *et al.*, 2005). Therefore, the detection of the phosphorylated form of AKT and S6 can represent mTOR activity as shown in Fig. 5J in our study, where mTOR activation is decreased under EHop‐016 treatment alone and combination treatment.

## 5 Conclusions

Our study demonstrates that RAC1 promotes ESCC progression and development and is associated with poor prognosis in patients. Inhibition of RAC1 reverses cisplatin resistance in ESCC both *in vivo* and *in vitro* via suppressing glycolysis. These findings outline the significance of RAC1 in regulating glycolysis and provide a novel insight into the mechanisms of chemoresistance in ESCC. Thus, RAC1 is a promising therapeutic target for the treatment of ESCC patients.

## Conflict of interest

The authors declare no conflict of interest.

## Author contributions

R‐JZ, C‐WZ, L‐YX, and E‐ML conceptualized the study; R‐JZ, C‐WZ, L‐YX, and E‐ML developed the methodology; R‐JZ, C‐WZ, J‐EG, H‐XZ, LX, L‐YX, and E‐ML carried out investigation; R‐JZ and C‐WZ wrote the original draft of the manuscript; J‐EG, H‐XZ, LX, and E‐ML reviewed and edited the manuscript; R‐JZ, C‐WZ, J‐EG, LX, L‐YX, and E‐ML acquired funding; R‐JZ, L‐YX, and E‐ML provided resources; and L‐YX and E‐ML supervised the study.

## Supporting information


**Fig. S1** **.** Combination therapy of cisplatin and RAC1 inhibitor suppresses chemoresistance to cisplatin in preventing ESCC cell proliferation.
**Fig. S2** **.** RAC1 inhibitor EHop‐016 inhibits ESCC cell viability.
**Fig. S3** **.** FOXO3a modulates the expression of glycolytic enzymes.

**Table S1** **.** Characteristics of the ESCC patients.
**Table S2** **.** The correlation between RAC1 and clinicopathological characteristics in ESCC (n = 106).
**Table S3** **.** Primers used in this study.
