# Supplementary material for: RAC1 inhibition reverses cisplatin resistance in esophageal squamous cell carcinoma and induces downregulation of glycolytic enzymes
Source: Mol Oncol. 2019 Jul 27;13(9):2010–30. doi: 10.1002/1878-0261.12548 (PMC6717762; doi:10.1002/1878-0261.12548)
Supplement: Supplementary file 1 — Fig. S1 . Combination therapy of cisplatin and RAC1 inhibitor suppresses chemoresistance to cisplatin in preventing ESCC cell proliferation. Fig. S2 . RAC1 inhibitor EHop‐016 inhibits ESCC cell viability. Fig. S3 . FOXO3a modulates the expression of glycolytic enzymes. [file MOL2-13-2010-s001.docx]

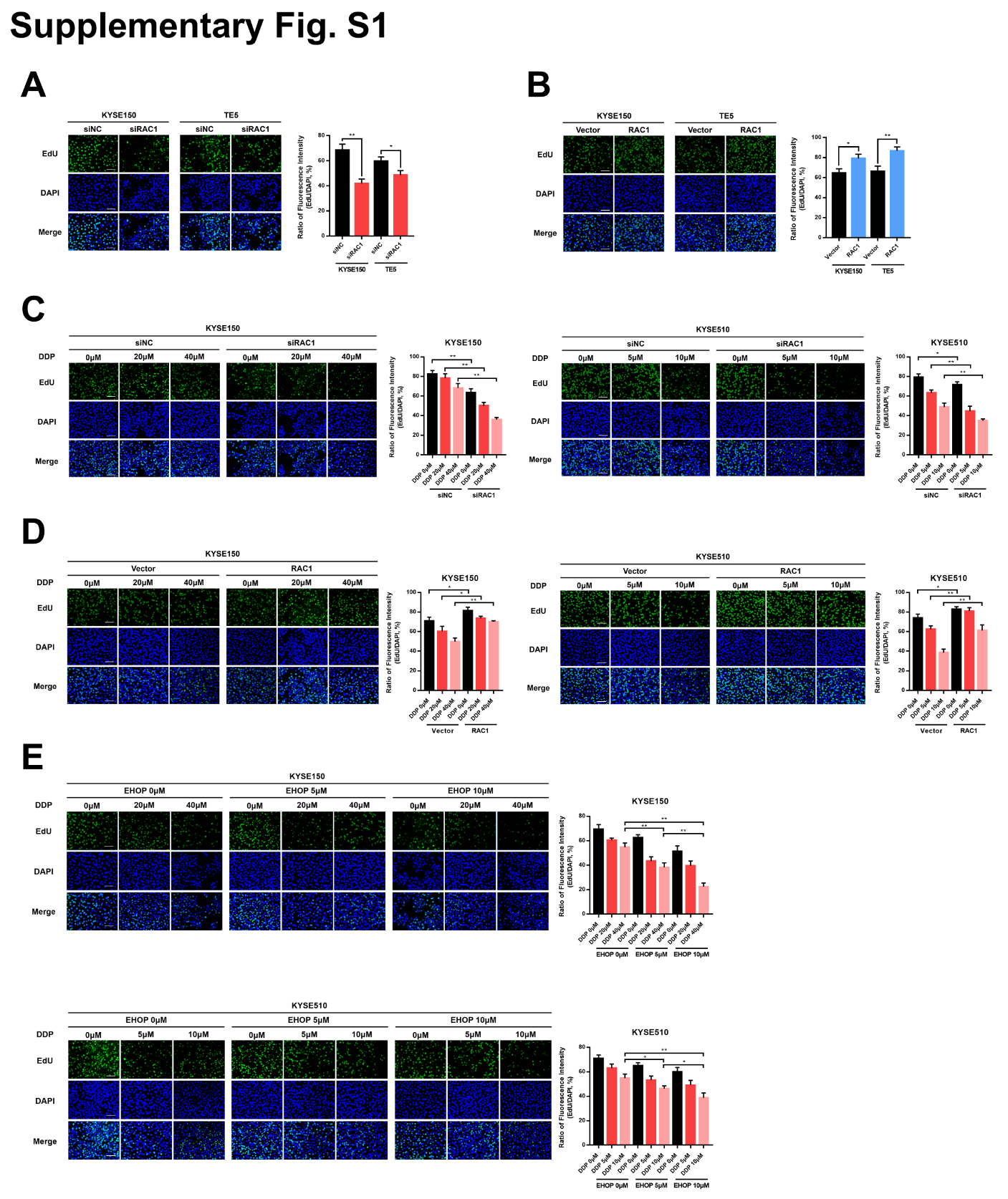


**Supplementary Figure S1. Combination therapy of cisplatin and RAC1 inhibitor suppresses chemoresistance to cisplatin in preventing ESCC cell proliferation.** A. KYSE150 and TE5 cell proliferation under RAC1-silencing was measured by EdU incorporation assay. B. KYSE150 and TE5 cell viability after RAC1-overexpression was measured by EdU incorporation assay. C. Proliferation of KYSE150 and KYSE510 cells that were treated with the combination of cisplatin and RAC1-silencing was determined by EdU incorporation assay. D. Viability of KYSE150 and KYSE510 cells that were treated with the combination of cisplatin and RAC1-overexpression was determined by EdU incorporation assay. E. KYSE150 and KYSE510 cell viability was detected after treatment of cisplatin and EHop-016 combination therapy. All scale bars, 100 μm. **P* < 0.05; ***P* < 0.01. Statistic differences in Fig. S1 were analyzed using *Student’s t-tests*. Error bars in Fig. S1 represent SD from triplicate experiments.

** Supplementary Figure S2. RAC1 inhibitor EHop-016 inhibits ESCC cell viability.** After being treated with different concentrations of RAC1 inhibitor EHop-016 (0μM, 5μM, 10μM, 15μM and 20μM for KYSE150; 0.0μM, 2.5μM, 5.0μM, 7.5μM and 10.0μM for KYSE510), MTS assay was performed to determine cell viability. Cell viability of KYSE150 and KYSE510 cells decreased accordingly to the increased concentration of EHop-016. Statistic differences in Fig. S2 were analyzed using *Student’s t-tests*. Error bars in Fig. S2 represent SD from triplicate experiments.

**Supplementary Figure S3. FOXO3a modulates the expression of glycolytic enzymes.** After transfection with siNC and siFOXO3a, Western blot was used to assess the cellular expression of P-FOXO3a, PKM, LDHA, ALDOA, and HK1. Knockdown of FOXO3a decreased the expression of P-FOXO3a, as well as the glycolytic enzymes PKM, LDHA, ALDOA and HK1 in KYSE150 and KYSE510 cells.
