# Supplementary material for: RAC1 inhibition reverses cisplatin resistance in esophageal squamous cell carcinoma and induces downregulation of glycolytic enzymes
Source: Mol Oncol. 2019 Jul 27;13(9):2010–30. doi: 10.1002/1878-0261.12548 (PMC6717762; doi:10.1002/1878-0261.12548)
Supplement: Supplementary file 2 — Table S1 . Characteristics of the ESCC patients. Table S2 . The correlation between RAC1 and clinicopathological characteristics in ESCC (n = 106). Table S3 . Primers used in this study. [file MOL2-13-2010-s002.docx]

| **Supplementary Table S1: Characteristics of the ESCC patients** | | |
| --- | --- | --- |
| **Clinical parameters** | **Total patient number (n=106)** | |
|  | **Patient number** | **Percentage** |
| **Gender** |  |  |
| Male | 79 | 75% |
| Female | 27 | 25% |
| **Age** |  |  |
| ≤58 years | 58 | 55% |
| >58 years | 48 | 45% |
| **Tumor Size** |  |  |
| <3cm | 29 | 27% |
| 3-5cm | 50 | 47% |
| >5cm | 27 | 25% |
| **Histologic grade** |  |  |
| G1 | 32 | 30% |
| G2 | 65 | 61% |
| G3 | 9 | 8% |
| **Primary tumor** |  |  |
| Tis | 0 | 0% |
| T1 | 8 | 8% |
| T2 | 11 | 10% |
| T3 | 86 | 80% |
| T4 | 1 | 1% |
| **Regional lymph node** |  |  |
| N0 | 69 | 65% |
| N1 | 23 | 22% |
| N2 | 14 | 13% |
| N3 | 0 | 0% |

| \| **Supplementary Table S2: The correlation between RAC1 and clinicopathological characteristics in ESCC (n=106)** \| \| \| \| \| \| \| --- \| --- \| --- \| --- \| --- \| --- \| \| **Variables** \| **RAC1 expression^1^** \| \| ***χ*²** \| **R** \| ***P**** \| \| **Low (n=39)** \| **High**  **(n=67)** \| \| **Age (year)** \|  \|  \| 0.558 \| 0.073 \| 0.915 \| \| <59 \| 25 \| 38 \|  \|  \|  \| \| ≥59 \| 14 \| 29 \|  \|  \|  \| \| **Gender** \|  \|  \| 0.799 \| -0.087 \| 0.820 \| \| Male \| 31 \| 48 \|  \|  \|  \| \| Female \| 8 \| 19 \|  \|  \|  \| \| **Tumor size** \|  \|  \| 3.147 \| 0.007 \| 0.045 \| \| <3cm \| 13 \| 16 \|  \|  \|  \| \| 3-5cm \| 14 \| 36 \|  \|  \|  \| \| >5cm \| 12 \| 15 \|  \|  \|  \| \| **Tumor location** \|  \|  \| 0.653 \| -0.015 \| 0.828 \| \| upper \| 2 \| 6 \|  \|  \|  \| \| middle \| 25 \| 39 \|  \|  \|  \| \| lower \| 12 \| 22 \|  \|  \|  \| \| **Histologic grade** \|  \|  \| 0.324 \| -0.006 \| 0.0827 \| \| G1 \| 12 \| 19 \|  \|  \|  \| \| G2 \| 23 \| 42 \|  \|  \|  \| \| G3 \| 4 \| 5 \|  \|  \|  \| \| **Primary tumor** \|  \|  \| 0.004 \| 0.006 \| 0.076 \| \| T1+T2 \| 7 \| 12 \|  \|  \|  \| \| T3+T4 \| 31 \| 55 \|  \|  \|  \| \| **Regional lymph node** \| \|  \| 0.645 \| -0.006 \| 0.001 \| \| N0 \| 26 \| 43 \|  \|  \|  \| \| N1 \| 7 \| 16 \|  \|  \|  \| \| N2 \| 6 \| 8 \|  \|  \|  \| \| *Fisher's Exact Test; *P*<0.05 was considered significant. \| \| \| \| \| \| \| ^1^Low ≤ 121 scores; High > 121 scores. \| \| \| \| \| \| |
| --- | --- | --- | --- | --- | --- | --- | --- | --- | --- | --- | --- | --- | --- | --- | --- | --- | --- | --- | --- | --- | --- | --- | --- | --- | --- | --- | --- | --- | --- | --- | --- | --- | --- | --- | --- | --- | --- | --- | --- | --- | --- | --- | --- | --- | --- | --- | --- | --- | --- | --- | --- | --- | --- | --- | --- | --- | --- | --- | --- | --- | --- | --- | --- | --- | --- | --- | --- | --- | --- | --- | --- | --- | --- | --- | --- | --- | --- | --- | --- | --- | --- | --- | --- | --- | --- | --- | --- | --- | --- | --- | --- | --- | --- | --- | --- | --- | --- | --- | --- | --- | --- | --- | --- | --- | --- | --- | --- | --- | --- | --- | --- | --- | --- | --- | --- | --- | --- | --- | --- | --- | --- | --- | --- | --- | --- | --- | --- | --- | --- | --- | --- | --- | --- | --- | --- | --- | --- | --- | --- | --- | --- | --- | --- | --- | --- | --- | --- | --- | --- | --- | --- | --- | --- | --- | --- | --- | --- | --- | --- | --- | --- | --- | --- | --- | --- | --- | --- | --- | --- | --- | --- | --- | --- | --- | --- | --- |

| **Supplementary Table S3: Primers used in this study** | | | | |
| --- | --- | --- | --- | --- |
| **Primer** | **Primer Sequence**  **(F: forward; R: reverse)** | **Annealing temperature (°C)** | | **Amplicon size (bp)** |
| ***PKM*** | F: AGCGGTCTTTGCTAGTGAGG  R: TGCTGAGGTCCTTTGGTTCTC | | 59 | 98 |
| ***LDHA*** | F: CATGGCCTGTGCCATCAGTA  R: AGATATCCACTTTGCCAGAGACA | | 60 | 158 |
| ***HK1*** | F: AGTGGAAGGAGCAGATGTGGTC  R: TGTAGCAAGCATTGGTGCCAGTG | | 62 | 179 |
| ***STAG1*** | F: GCCGGCCTCCATCTACAAAT  R: CACGGACTGCATTGCACTTT | | 60 | 182 |
| ***CCNB1*** | F: GCACTTCCTTCGGAGAGCAT  R: TGTAGAGTTGGTGTCCATTCAC | | 60 | 189 |
| ***MAD1L1*** | F: CAGGAACTACGAGCGTGAGG  R: GGGCCAGACTGTCCTCTTTC | | 60 | 182 |
| ***SERPINE*** | F: GCGCTGCAGAAAGTGAAGAT  R: AAGGACTGTTCCTGTGGGGT | | 59 | 156 |
| ***SESN2*** | F: CGCCACTCAGAGAAGGTCCA  R: TGGGACACAAAAGGGTCTGG | | 61 | 185 |
